# The quality of air outside and inside the home: associations with emotional and behavioural problem scores in early childhood

**DOI:** 10.1186/s12889-019-6733-1

**Published:** 2019-04-15

**Authors:** Emily Midouhas, Theodora Kokosi, Eirini Flouri

**Affiliations:** Department of Psychology and Human Development, UCL Institute of Education, University College London, 25 Woburn Square, London, WC1H 0AA UK

**Keywords:** Air pollution, Damp, Emotional and behavioural problems, Millennium Cohort Study, Secondhand smoke

## Abstract

**Background:**

This study explored the role of outdoor air pollution [nitrogen dioxide (NO_2_) and sulphur dioxide (SO_2_)] and indoor air quality (measured with damp or condensation and secondhand smoke exposures) at age 9 months in emotional, conduct and hyperactivity problems at age 3 years.

**Method:**

Data from 11,625 Millennium Cohort Study children living in England and Wales were modelled using multilevel regression.

**Results:**

After adjusting for a host of confounders, having a damp or condensation problem at home was related to both emotional and conduct problems. Secondhand smoke exposure was associated with all three problem types. Associations with outdoor air pollution were less consistent.

**Conclusions:**

Exposures to damp or condensation and secondhand smoke in the home are likely to be risk factors for child emotional and behavioural problems. Parents should continue to be educated about the dangers of exposing their children to poor air quality at home.

## Background

It has been recently estimated that outdoor air pollution results in around 40,000 deaths per year in the UK, predominantly caused by harmful toxins emitted by diesel engines such as nitrogen dioxide (NO_2_) and particulate matter (PM) [1]. Air pollution has been associated with a number of adverse respiratory and cardiovascular health outcomes in UK adults and children [2, 3]. Yet little is known about its impact on child mental health in the UK. About 10% of UK children are estimated to have severe mental health problems [4]. Such problems are associated with increased risk of mental disorders and crime in adulthood [5].

Exposure to air pollutants may increase children’s risk of poor mental health through its impact on the developing brain and by promoting inflammation. Evidence, from mainly animal but some human studies too, suggests that air pollution can lead to systemic inflammation that triggers endothelial activation and breakdown of the blood-brain barrier (BBB) [6]. Weakening of the BBB facilitates the passage of air pollutants into the brain via the BBB leading to neuroinflammation, the elevation of pro-inflammatory cytokines and reactive oxygen species. Knowledge of the exact mechanisms by which pollutants in the brain bring about neuroinflammation is still developing, although microglia, the immune response cells of the central nervous system, are likely to be integral to this process [7]. Also developing is research on the specific areas of the brain that may be affected. Studies by Calderón-Garcidueñas and colleagues [8–10] with children in Mexico City where air pollution levels are extremely high have found evidence that suggests that the prefrontal cortex, a region important for behavioural regulation and which undergoes considerable development throughout childhood and adolescence [11], may be particularly sensitive to neurological effects of air pollution. Other research studies have found elevated neuroinflammation in the hippocampus due to air pollutants [12].

The pollution-child mental health link via inflammation is as yet unexplored, despite much evidence for the link between pollution and inflammation and for the one between inflammation and mental health problems (although mainly in adult populations) [13, 14]. For example, studies have found higher levels of inflammatory markers including interleukin 6 (IL-6), a multifunctional cytokine, and C-reactive protein (CRP), an acute phase protein, among clinical patients with psychiatric disorders [15, 16] compared with individuals without these disorders. Possible mechanisms linking inflammatory responses in the central nervous system to mental health problems including depression and anxiety are hyperactivity and hypoactivity of the hypothalamic-pituitary-adrenal axis, oxidative stress and altered monoamine and glutamate neurotransmission and hippocampal neurogenesis (evidenced in adults, but unexplored in children) [13, 17–19].

What research *has* established is that children exposed to air pollutants, either in the prenatal or in the postnatal period, are at greater risk of behavioural problems, a diagnosis of attention deficit/hyperactivity disorder and being prescribed medication for psychiatric disorders [17, 20–28]. For example, one study in the U.S. found a relationship between exposures to black carbon attributed to traffic in the first year of life and children’s symptoms of hyperactivity at age 7 in the Cincinnati Childhood Allergy and Air Pollution Study birth cohort [29]. However, the relationship applied only to children exposed to the highest tertile of traffic-related pollution and who had a mother with more than a secondary school degree.

Another, by Harris et al. [21], using data from a small pregnancy cohort in Massachusetts, U.S., identified a link between black carbon exposure and behavioural problems as reported by teachers. A third, by Oudin et al. [23] in Sweden, found effects of NO_2_ and PM on dispensing of medication for child and adolescent mental health problems. More recently, Min and Min [22] identified an association between NO_2_ and a diagnosis of attention deficit/hyperactivity disorder in a large sample of Korean children tracked from birth until age 10. Studies on non-human subjects also show adverse effects. Experimental studies exposing laboratory animals (rats and mice) to polycyclic aromatic hydro-carbon during the postnatal period have reported anxiety and depression-like symptoms [30, 31].

Estimating the genuine impact of outdoor air pollution on child mental health outcomes is not straightforward. The majority of studies do not account for sources of indoor air pollution that may be confounders [29, 32]. Also, children spend most of their time indoors and, as such, exposure to indoor pollutants generated from activities such as heating, cooking, smoking and use of cleaning products, varnishes and paints may be more important for young children than exposure to outdoor pollutants. Moreover, outdoor air quality may affect indoor air quality as pollutants from outdoors (e.g., produced by traffic emissions and industrial processes) may enter the home through windows or other types of ventilation. Therefore, alongside indicators of outdoor air quality two measures of indoor air quality were considered in this study: damp or condensation and secondhand smoke.

Damp or condensation can cause growth of moulds and bacteria that generate microscopic airborne particles [33], some of which contain allergens or chemicals that have the potential to cause neuroinflammation. Whereas many epidemiological studies have uncovered associations between indoor mould and respiratory problems in children [34–38], studies addressing relationships between mould and mental health in childhood are rare [17, 39]. In fact, this is the first study, to our knowledge, that explores damp or condensation problems in the home and young children’s behaviour in a general population sample.

Secondhand smoke, the other indicator of indoor air quality we explored, has high concentrations of many toxic chemicals that are harmful to the brain [40]. For example, carbon monoxide in the bloodstream can reduce oxygen in the brain [41] and nicotine can impact the cholinergic system that regulates higher-order processing and memory [42, 43]. There is indeed evidence that preschool children exposed to secondhand smoke, either prenatally or postnatally, show more externalising (behavioural) problems than those unexposed [44–51], and that effects persist into adolescence [45, 49, 51].

There is therefore evidence supporting the link between poor air quality and child behaviour, but the existing research has several limitations that this study was carried out to address. First, although recent research has explored outdoor and indoor air quality and cognitive ability in early childhood [52], no study has explored simultaneously the roles of outdoor and indoor air quality in emotional and behavioural problems during this developmental phase. Second, no study has yet explored the associations between air quality and child emotional and behavioural problems in the general population (the research to date has targeted urban samples). Last, to our knowledge, no study on outdoor air pollution effects on child behaviour has adjusted for the amount of greenery in the child’s neighbourhood. Green space may confound the association between outdoor air pollution and child behaviour since lower pollution is likely to be found in greener neighbourhoods. With more land devoted to greenery, there is less land available for pollution-generating processes and combustion. Additionally, trees (one of the two key components of urban vegetation) capture large amounts of airborne gaseous, particulate and aerosol pollutants, thereby reducing their concentration in the air [53–57]. Therefore, woodlands and the presence of trees and, in general, amount of green space in the environment can improve air quality, and are, at the same time, negatively related to emotional problems among children [58, 59].

The present study aimed to address these gaps in the literature through a secondary analysis of data from the UK’s Millennium Cohort Study (MCS), a longitudinal study of 19,519 children, born around 2000, and their families. It explored the association between neighbourhood-level (i.e. ward^1^) NO_2_ and sulphur dioxide (SO_2_) measured at the beginning of MCS (at age 9 months) and child emotional and behavioural problems at age 3 (when first measured in MCS) in England and Wales. Although brain development continues well into adulthood, the first 2–3 years of life is a key period for brain development when children may be particularly vulnerable to the impact of environmental toxins [60].

NO_2_ and SO_2_ are  gaseous pollutants mainly caused by fossil fuel combustion [61]. SO_2_ is an acid gas resulting from the combustion of fossil fuels that contain sulphur such as coal and heavy oils. NO_2_ results from nitrogen oxides reacting with oxygen or ozone. Road traffic (mainly from diesel emissions) and energy production processes are the main sources of NO_2_. The EU has published air quality standards indicating the legal levels of pollutants, including those outlined in the EU Ambient Air Quality Directive and the fourth Daughter Directive. In the UK, the level of NO_2_ has regularly exceeded the legal level. In 2013, around one-third of licensed cars in the UK were diesel [62]. As a result, air pollution exposures have become an important concern for policymakers, urban planners and citizens, especially in large urban areas (e.g., London) where pollution levels are highest. Hence, understanding the effects of air pollution on children may have important implications for UK transport and environmental policy as well as urban planning. Unfortunately, UK air quality standards presently apply to outdoor air only and not to indoor air in residential homes. With regard to indoor air quality, the Committee on the Medical Effects of Air Pollutants (COMEAP) have published guidelines [63] and the National Institute for Health and Care Excellence (NICE) and Public Health England (PHE) are presently developing guidelines to be published in 2019 (https://www.nice.org.uk/guidance/indevelopment/gid-ng10022). It is important that we continue to further our understanding of the risks posed by both outdoor and indoor air pollutants, to inform policies and practices that affect outdoor air quality as well as the development of standards for indoor air quality in homes.

## Methods

### Study sample

The Millennium Cohort Study (MCS) is a longitudinal survey taking its sample from all births in the UK over a year, starting on 1 September 2000 [64]. There have been six sweeps of data thus far with 19,519 children participating in at least one of these sweeps. The sample is disproportionately stratified to allow for sufficient numbers in the four UK countries and electoral wards with disadvantaged or (in England) ethnic minority populations [64]. Ethical approval was gained from National Health Service Research Ethics Committees and parents gave written informed consent for themselves and their children before interviews took place, at each sweep.

We used data^2^ from Sweeps 1 (at age 9 months, dates ranging June 2001 to January 2003) and 2 (at 3 years, dates ranging September 2003 to April 2005). Initially, we retained singleton children and first born twins/triplets (19,244 out of 19,519), making the number of children equal to the number of families. Our ‘analytic’ sample (those included in our analyses) included children living in England or Wales and who had data on emotional and behavioural problems, at Sweep 2 (*n* = 11,625). Our ‘non-analytic’ sample (those not included in our analyses) comprised children who did not live in England and Wales and/or did not have data on emotional and behavioural problems, at Sweep 2, i.e., at age 3 (*n* = 7619).

### Measures

Emotional and behavioural problems at age 3 were measured with 15 items (on 3-point response scales) of the parent-reported Strengths and Difficulties Questionnaire (SDQ) [65] measuring hyperactivity (α = .71 across sweeps), emotional symptoms (α = .52) and conduct problems (α = .69). Each of the three scales has 5 items. Examples of items from the hyperactivity scale are ‘restless, overactive, cannot stay still for long’ and ‘constantly fidgeting or squirming’. Emotional symptoms items include ‘many worries, often seems worried’ and ‘often unhappy, down-hearted and tearful’, and examples of conduct problems items are ‘often fights with other children and bullies them’ and ‘often has temper tantrums or hot tempers’. Each SDQ item is scored 0 if the response is ‘not true’, 1 for ‘somewhat true’ and 2 for ‘certainly true’. Scores for each scale may range 0–10. In our sample, internal consistency (see above) was in line with other research using the SDQ [66].

Outdoor air pollutants,^3^ SO_2_ and NO_2_, were measured at age 9 months with data from the Multiple Environmental Deprivation Index (MEDIx). MEDIx is a measure of physical environmental deprivation that represents both pathogenic and salutogenic characteristics in each UK ward [67, 68]. Air pollution was measured with annual mean concentrations [in micrograms (one-millionth of a gram) per cubic meter air or μg/m^3^] within each ward. Means are population weighted using output area (OA) units, and cover the years 1999–2003. Data were taken from 1 km grids, modelled from National Atmospheric Emissions Inventory data. The annual mean values were converted to deciles across all wards in the UK. In the bottom decile the annual mean concentration of NO_2_ is less than 9.26 μg/m^3^ and in the top decile it is greater than 33.89. Note that the annual mean legal limit for NO_2_ is 40 μg/m^3^ [69]. With regard to SO_2,_ these values are 1.78 and 7.24, respectively. The annual mean legal limit for SO_2_ is 20 μg/m^3^.

Indoor air quality was measured by the mother’s report of whether there was a *damp or condensation* problem at home and whether the baby was exposed to *secondhand smoke,* both at age 9 months. For the latter, mothers were asked whether anyone smokes in the same room as the baby.

We also measured two additional neighbourhood factors at age 9 months – urbanicity and the percentage of green space. *Urbanicity* was measured with a variable that indicated if the family resided in an urban area (i.e., a settlement with a population greater than 10,000) or not. The percentage of *green space*, covering all green spaces larger than 5 m^2^ (excluding private gardens) for each ward, was measured with data from the MEDIx. Green spaces include neighbourhood greenery, parks, playing fields and forests/woodlands. Estimates are based on land use data from the Generalised Land Use Database [70] and the Coordination of Information on the Environment [71]. In MCS, the percentages of ward-level greenery using the MEDIx data are available in deciles across all wards in the UK. The lowest decile corresponds to wards with 0–21% green space and the top decile refers to wards with 94–95% green space.

A range of key family and child covariates were included. Family-level variables (measured at age 3) were household income (equivalised weekly income, adjusting for household size and composition), maternal education (University degree or not), maternal psychological distress, measured with the Kessler Psychological Distress scale (K6), a 6-item screener of psychological distress [72] and maternal general health (self-reported). For the latter, a binary variable was used where ‘good’ or ‘excellent’ general health was compared to ‘fair’ or ‘poor’. The child-level covariates were gender, age (in years), ethnicity (White, Black, Indian, Pakistani/Bangladeshi, Mixed and Other) and low birth weight (< 2.5 kg).

### Statistical analysis

The main aim of the study was to examine the relationship between outdoor and indoor air quality at age 9 months and emotional and behavioural problems at age 3, controlling for neighbourhood greenery and urbanicity, family-level variables related to neighbourhood selective sorting and emotional and behavioural problems and child covariates. To meet this aim, we first carried out descriptive analyses, including correlations among the main variables of the study. As MCS data have a hierarchical structure due to the sampling design (with children/families nested within areas), we fitted 2-level linear regression models for each SDQ scale we considered (emotional problems, conduct problems and hyperactivity problems). We accounted for MCS area clustering at the ward-level and, as explained, the environmental variables (outdoor air pollution, green space and urbanicity) were measured at ward-level, thus aligning with the clustering of cohort families in wards in MCS.

In our models, we adjusted for the MCS strata to reflect the stratified sample design: in each of the four UK countries, families were oversampled from areas with high child poverty (‘disadvantaged’) and (in England only) from areas with high proportions of ethnic minorities (‘ethnic’). Since our sample included only children from England and Wales, we adjusted for five strata: 1) England-advantaged, 2) England-disadvantaged, 3) England-ethnic, 4) Wales-advantaged and 5) Wales-disadvantaged. Listwise deletion was used in all models.

## Results

On average, children in the analytic sample lived in wards in England and Wales with NO_2_ and SO_2_ annual concentrations at the higher end of the distribution of wards in the UK (Table 1). Around 14% and 12% families in the analytic sample reported damp or condensation and secondhand smoke in the home, respectively. Additionally, they resided in comparatively less green neighbourhoods across the distribution of wards in the UK. The majority of the sample lived in urban areas (83%). With regard to emotional/behavioural problems, SDQ scores ranged 1–4 depending on the domain, on average, which is at the lower end of the 0–10 range. There were statistically significant correlations (Table 2), as expected, across the main study variables. The associations between air quality (both indoor and outdoor) and emotional and behavioural problems, however, were weak.

**Table 1:** Descriptives of study variables in the analytic sample (*n* = 11,625)

	*n*	*%*
Child		
Female	5706	49.26
*Ethnicity*		
White	9550	86.65
Black	424	2.90
Indian	333	1.89
Pakistani/Bangladeshi	720	3.67
Mixed	420	3.62
Other	173	1.26
Low birth weight	794	6.83
Parent/household (S2)		
Mother is university-educated	3748	33.69
Mother has good general health	9371	81.52
Indoor air quality (S1)		
Damp or condensation problem	1598	14.22
Secondhand smoke	1397	12.36
Neighbourhood (S1)		
Urbanicity	9336	83.01
	***n***	***M (SD)***
Child (S2)		
Emotional problems	11,549	1.36 (1.41)
Conduct problems	11,569	2.84 (1.98)
Hyperactivity problems	11,463	3.97 (2.26)
Age (years)	11,625	3.13 (0.19)
Parent/household (S2)		
Household income	10,924	324.01(196.36)
Maternal psychological distress	9660	3.28 (3.50)
Neighbourhood (S1)		
NO_2_ deciles	11,035	7.27 (2.27)
SO_2_ deciles	11,035	6.51 (2.34)
Green space deciles	11,035	4.27 (2.33)

*Note*: *S1* Sweep 1 (age 9 months), *S2* Sweep 2 (age 3 years). Estimates are weighted and Ns are unweighted

**Table 2:** Correlations (Pearson’s *r* coefficients) among outdoor air pollutant concentrations and indoor air quality at age 9 months and emotional/behavioural problems at age 3 years in the analytic sample (n = 11,625)

Variables	Emotional symptoms	Conduct problems	Hyperactivity	SO_2_ (deciles)	NO_2_ (deciles)	Damp/condensation	Secondhand smoke
Emotional symptoms	1						
Conduct problems	.31***	1					
Hyperactivity	.25***	.49***	1				
SO_2_ (deciles)	.05***	.08***	.08***	1			
NO_2_ (deciles)	.07***	.04**	.06***	.45***	1		
Damp/condensation	.07***	.09***	.06***	.01	.06***	1	
Secondhand smoke	.10***	.18***	.14***	.05***	−.02*	.07***	1

Note: *p < .05, **p < .01, ***p < .001

With respect to selection bias, the children in the analytic sample (*n* = 11,625) differed from those in the non-analytic sample (*n* = 7619) on all variables except for gender, maternal education and birth weight. For example, the children in the analytic sample were more likely to have higher household incomes (*p* < .001) and to have mothers with more psychological distress (p < .001). The analytic sample was additionally more likely to live in areas with higher levels of SO_2_ (p < .001) and NO_2_ (p < .001) and less green space (p < .001). It also had proportionately fewer cases of secondhand smoking exposure (p < .001) but more cases of exposure to damp or condensation in the home (p < .001) and more emotional, conduct and hyperactivity problems.

Exposures to NO_2_ and SO_2_ in infancy were not significantly associated with emotional symptoms or hyperactivity at age 3 (Table 3). However, an increase in one decile across the distribution of wards by NO_2_ in infancy was significantly associated with an increase in conduct problems at age 3, even after adjusting for SO_2_ levels, indoor air quality, neighbourhood green space, urbanicity and family factors associated with area selection. However, the (unstandardised) coefficient for conduct problems was small (b = 0.03).

**Table 3:** Effects of indoor and outdoor air quality at age 9 months on emotional, conduct and hyperactivity problems at age 3 years

	Emotional symptoms		Conduct problems		Hyperactivity problems	
	Coeff. (SE)	95% CI	Coeff. (SE)	95% CI	Coeff. (SE)	95% CI
Fixed effects						
Constant	0.90**(0.26)	[0.39, 1.41]	3.70***(0.356)	[3.00, 4.39]	3.96***(0.41)	[3.15, 4.77]
SO_2_ (deciles)	0.004(0.01)	[−0.01, 0.02]	0.01(0.01)	[− 0.01, 0.03]	0.02(0.01)	[− 0.001, 0.05]
NO_2_ (deciles)	0.01(0.01)	[− 0.01, 0.02]	0.03*(0.01)	[0.004, 0.06]	0.02(0.02)	[−0.01, 0.50]
Damp or condensation	0.09*(0.04)	[0.002, 0.17]	0.16**(0.06)	[0.04, 0.27]	0.03(0.07)	[−0.10, 0.17]
Secondhand smoke	0.16**(0.05)	[0.07, 0.25]	0.59***(0.06)	[0.47, 0.71]	0.37***(0.07)	[0.23, 0.52]
Green space	0.01(0.01)	[−0.01, 0.02]	0.04**(0.01)	[0.02, 0.07]	0.02(0.02)	[−0.01, 0.05]
Urban	0.07(0.05)	[−0.04, 0.18]	0.08(0.08)	[−0.07, 0.23]	0.003(0.09)	[−0.17, 0.18]
Age	0.08(0.07)	[−0.06, 0.22]	−0.36***(0.10)	[− 0.55, − 0.16]	0.19(0.12)	[−0.04, 0.41]
Female	0.003(0.03)	[−0.06, 0.06]	−0.22***(0.04)	[− 0.30, − 0.15]	−0.60***(0.05)	[− 0.70, − 0.51]
* Ethnicity (Ref: White)*						
Mixed	−0.04(0.08)	[− 0.20, 0.13]	0.03(0.11)	[− 0.19, 0.26]	−0.01(0.13)	[− 0.27, 0.25]
Indian	0.26*(0.11)	[0.04, 0.48]	−0.02(0.16)	[−0.32, 0.29]	0.16(0.18)	[−0.20, 0.51]
Pakistani/Bangladeshi	0.81***(0.09)	[0.63, 0.99]	−0.03(0.13)	[−0.28, 0.21]	0.32*(0.15)	[0.03, 0.61]
Black	−0.11(0.10)	[−0.31, 0.08]	− 0.32*(0.13)	[− 0.58, − 0.06]	−0.28(0.16)	[− 0.58, 0.03]
Other	0.23(0.18)	[−0.12, 0.58]	−0.32(0.24)	[− 0.79, 0.15]	−0.32(0.29)	[− 0.88, 0.24]
Low birth weight	0.18**(0.06)	[0.06, 0.30]	0.04(0.09)	[−0.13, 0.21]	0.53***(0.10)	[0.33, 0.72]
Mother is university-educated	−0.16***(0.03)	[−0.23, − 0.09]	−0.31***(0.05)	[− 0.40, − 0.22]	−0.48***(0.06)	[− 0.59, − 0.37]
Household income	−0.00***(0.00)	[− 0.00, − 0.00]	−0.001***(0.00)	[− 0.001, − 0.001]	−0.001***(0.00)	[− 0.001, − 0.001]
Maternal psychological distress	0.08***(0.00)	[0.07, 0.09]	0.13***(0.01)	[0.12, 0.14]	0.11***(0.01)	[0.10, 0.12]
Mother has good general health	−0.17***(0.04)	[−0.25, − 0.10]	−0.25***(0.06)	[− 0.36, − 0.15]	−0.22**(0.06)	[− 0.34, − 0.09]
Random effects						
Ward-level intercept variance (Level 2)	0.00(0.00)	[0.00, 0.00]	0.01(0.01)	[0.00, 0.09]	0.003(0.01)	[0.00, 3.55]
Child-level intercept variance (Level 1)	1.93***(0.03)	[1.87, 1.99]	3.58***(0.05)	[3.48, 3.69]	4.87***(0.07)	[4.73,5.02]

Notes. *p < .05; **p < .01; ***p < .001. Coefficients are unstandardised. All models are adjusted for the area strata

The two measures of indoor air quality in infancy were significantly related to both emotional and conduct problems but only secondhand smoke exposure was related to hyperactivity problems (b = 0.37; Table 3). With regard to conduct problems, damp/condensation in the home and exposure to secondhand smoke were significantly associated with an increase of 0.16 and 0.59 problems, respectively. Damp/condensation in the home and exposure to secondhand smoke were related positively to emotional problems (b = 0.09 and b = 0.16, correspondingly).

With respect to the other variables in the model, urbanicity was not significantly associated with any problem type. The amount of green space was significantly related to conduct problems (b = 0.04) but not to emotional problems or hyperactivity. Household income and mother’s university education and good general health were significantly associated with fewer problems in all three domains. With regard to the child factors, Indian and Pakistani/Bangladeshi (relative to White) children as well those with low birth weight had more emotional problems. Girls and children of Black ethnic origin (relative to White) had fewer conduct problems. Girls were less likely to have hyperactivity problems and children from Pakistani/Bangladeshi backgrounds as well as those with low birth weight were more likely to have such problems. Random effects for all models showed that the between-child variance in emotional, conduct and hyperactivity problems was significant, but the between-ward variance in children’s problems was not.^4^

## Discussion

This study assessed the role of outdoor neighbourhood exposure to NO_2_ and SO_2_ and indoor residential exposure to damp/condensation and secondhand smoke at age 9 months in three domains of child behaviour – emotional symptoms, conduct problems and hyperactivity problems (measured at age 3 years) – in a nationally representative sample in England and Wales. A range of important confounders were included such as neighbourhood greenery and urbanicity as well as family background characteristics related to selective sorting into neighbourhoods.

We found that our indoor air quality indicators were associated with multiple child behaviour outcomes. Living in a home with a damp or condensation problem at age 9 months was related to both emotional symptoms and conduct problems at age 3. Effects were robust to adjustment for socio-economic status including maternal education and household income as well as measures of outdoor air quality including green space and air pollution. This is the first study we are aware of to link indoor damp/condensation to child behaviour and mental health in a general population sample. Moreover, secondhand smoke exposure at age 9 months was related to all three child outcomes at age 3, with the largest effect seen for conduct problems (equivalent to an increase in .59 points on the conduct problems scale). Most of the extant literature has examined the relationship between prenatal rather than postnatal exposure to smoking and child behaviour [44–46, 49, 50]. However, reflecting this study’s finding, a fairly recent study in Scotland found that exposure to secondhand smoke (objectively-measured) was associated with poorer child behaviour, particularly externalising problems, also measured with the SDQ [47].

The probability of incorrectly rejecting the null hypotheses that there are no differences in behaviour for children living in homes with secondhand smoke and a damp or condensation problem is less than 5%, given we set our significance level at .05. However, at the same time, having such a large sample (*n* = 11,625) raises the likelihood of detecting significant effects in the sample. Thus, it is especially important to consider the practical significance of the effects detected. For example, considering the weaker effects we found for a damp or condensation problem (relative to secondhand smoke), the estimated differences in problems for a child with and without a damp or condensation problem were .09 and .16 points on the emotional symptoms and conduct problems scales, respectively. Note that each scale score ranges 0 to 10 and an increase in one point is the difference between a response of ‘not at all true’ and ‘somewhat true’ or a response of ‘somewhat true’ and ‘certainly true’. Therefore, an increase in .09 and .16 points is not even close to reaching the endorsement of a problem. On the other hand, the stronger effects on externalising problems for secondhand smoke were .37 (hyperactivity) and .59 (conduct problems). These reflect a difference of roughly a third and over a half of a problem respectively for those exposed and those unexposed to secondhand smoke. Still, explanatory variables in general were found to have small to moderate effect sizes. Nevertheless, our indoor air quality effects were akin to or greater than those found for other child and family factors known to be important for child behaviour. For example, in our models, a greater increase in conduct problems was found for children who were exposed to secondhand smoke than children whose mothers did not have a university degree. Therefore, we believe it is important to consider indoor air quality as another risk factor for child behaviour, alongside family background and key child characteristics, assuming the differences we have found are not due to sampling error and are true in the population.

Conversely, we did not find evidence for a reliable association between child behaviour and exposure to outdoor NO_2_ or SO_2._ The level of NO_2_ (but not SO_2_) in the neighbourhood at age 9 months had a very small but significant linear association only with conduct problems at age 3. This finding reflects those of Min and Min [22], Oudin et al. [23] and Newman et al. [29]. However, two of these studies [22, 23] found associations with clinical outcomes - diagnoses of attention deficit/hyperactivity disorder and dispensing of medication, respectively - rather than the continuum of problem behaviour explored in this study. Newman et al. [29] did find an association between exposures to high levels of traffic-related pollution in the first year of life (when we measured NO_2_) and symptoms of hyperactivity symptoms at age 7 (we examined child behaviour at age 3) but only for children whose mothers had attended higher or further education. That study also examined, but did not find, an association with conduct problems. Our study shows that even changes in externalising behaviour outside of a clinical range may result from air pollution exposures. Nonetheless, we must be cautious about the implications given the lack of effects for emotional problems and hyperactivity and the small effect size for conduct problems. The study’s findings may have implications for environmental policy with regard to indoor air quality, however. They may offer support for the need to develop UK health-based indoor air quality standards, especially about damp, condensation, mould and tobacco use.

Nonetheless, this study has several limitations. First, our findings regarding neighbourhood-level pollution may be due to traffic noise, neglected in other similar studies as well [22, 23, 29], which we could not adjust for in this study. Second, we could not consider exposures to pollution, smoking and alcohol prenatally [73] as MCS does not have data on prenatal exposures. Hence, the relationships we found between postnatal exposures and child behaviour may be at least partly due to exposures in pregnancy. Third, our measure of air pollution for each neighbourhood was an average of the annual mean concentrations across a 4-year period. The 4-year average may overlook annual changes in pollutant levels. Fourth, a smaller spatial resolution would have captured the more immediate outdoor air pollutants near children’s homes. A more appropriate neighbourhood geography might have been Lower layer Super Output Area (LSOA) or Output Area (OA) rather than ward. OAs are built from groups of adjacent postcodes in the UK (with an average of 309 residents) and are the base unit for census data releases. LSOAs are built from clusters of OAs (typically 4–6) with a population averaging 1500. Fifth, we were also unable to account for outdoor pollutants that may come from adjacent neighbourhoods. Sixth, we could not measure directly the amount of pollution indoors (e.g., NO_2_, PM) including those resulting from secondhand smoke and damp or condensation or from activities such as heating, cooking, and use of cleaning products, paints and varnishes. Relatedly, our measure of secondhand smoke in the home did not consider other sources such as parents or other residents wearing smoke-laden clothes inside the home. Finally, we were unable to measure whether reports of damp or condensation were provided during the UK condensation season (March to October) which may affect the extent of dampness or condensation in the home. Future research studies should endeavour to address these limitations.

## Conclusions

In a large UK general population sample, we found that exposures to damp or condensation and secondhand smoke in the home are likely to be risk factors for emotional and behavioural problems in young children, measured using a well-validated measure of emotional and behavioural problem scores. Thus, this study offers reasons to continue to educate parents about the health risks of exposing their children to poor air quality, particularly within the home, and may give further evidence for the need for health-related UK residential air quality standards. Future research should continue to explore the proposed mechanisms linking air pollution to child behaviour and mental health [74] including neuroinflammation via oxidative stress, changes in serotonin and glutamate neurotransmission and hyperactivity and hypoactivity of the hypothalamic-pituitary-adrenal axis [13, 18, 19] as well as identify which regions of the brain, that are implicated in mental health problems, are most affected by these consequences of neuroinflammation.

## Abbreviations

BBB: Blood-brain barrier
CO: Carbon monoxide
CRP: C-reactive protein
IL-6: Interleukin 6
K6: Kessler Psychological Distress 6-item scale
MCS: Millennium Cohort Study
MEDIx: Multiple Environmental Deprivation Index
NO_2_: Nitrogen dioxide
OA: Output area
PM: Particulate matter
SDQ: Strengths and Difficulties Questionnaire
SO_2_: Sulphur dioxide

## Footnotes

1 Electoral wards are the key building block of UK electoral geography (http://www.ons.gov.uk/ons/guide-method/geography/beginner-s-guide/administrative/england/electoral-wards-divisions/index.html). The average population is around 5500, though counts can vary substantially. Census area statistical (CAS) wards in particular are used in this study. They are a type of ward created for the 2001 census output produced by aggregating smaller output areas (http://webarchive.nationalarchives.gov.uk/20160108225108/http://www.ons.gov.uk/ons/guide-method/geography/beginner-s-guide/administrative/england/electoral-wards-divisions/statistical-wards%2D%2Dcas-wards-and-st-wards/index.html). In England and Wales, there were a total of 8850 CAS wards.

2 Note that all measures and exclusions for our study are presented in this paper.

3 Initially, we explored the correlations of all four pollutants available in MEDIx – particulate matter 10 μm or less in diameter (PM10), NO_2_, SO_2_ and carbon monoxide (CO) - in our sample. Correlations among PM10, NO_2_ and CO were high enough (.83–.91) to warrant including only one of these in a regression model to avoid problems with multicollinearity. SO_2_ was only moderately correlated with the other three pollutants (with correlations ranging .42–.46). Therefore, we decided to retain NO_2_ and SO_2_ only for the analysis.

4 We also considered whether the child’s health status might explain these differences in emotional and behavioural problems according to air pollution exposures. At age 3, parents were asked to report on whether their child had a longstanding illness or disability. We added this covariate to the regression models and the significance of the coefficients did not change.
